# Assessing Utilization and Environmental Risks of Important Genes in Plant Abiotic Stress Tolerance

**DOI:** 10.3389/fpls.2016.00792

**Published:** 2016-06-24

**Authors:** Mohammad S. Khan, Muhammad A. Khan, Dawood Ahmad

**Affiliations:** ^1^Faculty of Crop Production Sciences, Institute of Biotechnology and Genetic Engineering, The University of Agriculture, PeshawarPakistan; ^2^Research School of Biology, ANU College of Medicine, Biology and Environment, The Australian National University, Canberra, ACTAustralia

**Keywords:** abiotic stresses, transgenic plants, *codA*, DREBs, antiporters, biosafety assessment

## Abstract

Transgenic plants with improved salt and drought stress tolerance have been developed with a large number of abiotic stress-related genes. Among these, the most extensively used genes are the glycine betaine biosynthetic *codA*, the DREB transcription factors, and vacuolar membrane Na^+^/H^+^ antiporters. The use of *codA*, DREBs, and Na^+^/H^+^ antiporters in transgenic plants has conferred stress tolerance and improved plant phenotype. However, the future deployment and commercialization of these plants depend on their safety to the environment. Addressing environmental risk assessment is challenging since mechanisms governing abiotic stress tolerance are much more complex than that of insect resistance and herbicide tolerance traits, which have been considered to date. Therefore, questions arise, whether abiotic stress tolerance genes need additional considerations and new measurements in risk assessment and, whether these genes would have effects on weediness and invasiveness potential of transgenic plants? While considering these concerns, the environmental risk assessment of abiotic stress tolerance genes would need to focus on the magnitude of stress tolerance, plant phenotype and characteristics of the potential receiving environment. In the present review, we discuss environmental concerns and likelihood of concerns associated with the use of abiotic stress tolerance genes. Based on our analysis, we conclude that the uses of these genes in domesticated crop plants are safe for the environment. Risk assessment, however, should be carefully conducted on biofeedstocks and perennial plants taking into account plant phenotype and the potential receiving environment.

## Introduction

Abiotic stresses such as salt, drought and extreme temperatures are serious threats to agriculture, and account for more than 50% of average yield losses for most of the major crop plants worldwide (Vahdati and Leslie, 2013). Abiotic stresses induce changes at the morphological, physiological, biochemical and molecular level that adversely affect plant growth and productivity (Fraire-Velázquez and Balderas-Hernández, 2013). Salt and drought stresses, in particular, exert adverse effects on plant physiology and developmental processes mainly by disrupting the ionic and osmotic homeostasis (Smirnoff, 1998; Ruan and Teixeira da Silva, 2011). In response to these stress conditions, plants induce signal perception, signal transduction and expression of stress-related genes which lead to changes in metabolic processes (Rensink et al., 2005; Ruan and Teixeira da Silva, 2011).

With progress in identification of genome sequence information and tools for functional genomics, several crop plants have been engineered to enhance their abiotic stress tolerance (Blumwald, 2003; Peleg et al., 2011). Particularly, the genes encoding transcription factors, ion transporters and enzymes of osmoprotectants biosynthetic pathways have been used in transgenic plants to enhance their tolerance to multiple abiotic stresses (Vinocur and Altman, 2005; Bhatnagar-Mathur et al., 2008; Peleg et al., 2011).

In addition to the efforts to improve stress tolerance, addressing environmental concerns over the use of these genes in transgenic plants remains a debatable issue for future deployment and commercialization. There is wide consensus that the current risk assessment procedures are equally applicable to more complex traits such as abiotic stress tolerance (Wolt, 2009; Sammons et al., 2014). However, based on the more complex nature of abiotic stress tolerance trait, further investigations are needed to target the plant phenotype, the magnitude of stress tolerance and potential impact on non-target environment. An important consideration in the environmental risk assessment is to check whether extra measures are required for these genes and the conferred trait. In the present study, we review the need for further considerations based on properties of proteins encoded by these genes, underlying mechanisms, phenotype of the transgenic plant and the potential receiving environment.

## Utilization of Important Genes in Plant Abiotic Stress Tolerance

### Glycine Betaine and the *codA* Gene

Glycine betaine (GB) is one of the most important osmoprotectants that provides protection to vital cellular organelles during plant adaptation to abiotic stress (Bohnert et al., 1995). The protective role of GB has been demonstrated in a number of transgenic plants engineered with genes involved in various biosynthetic pathways of GB (Chen and Murata, 2008; Khan et al., 2009, 2015b). Among all genes of the GB biosynthetic pathway, the *codA* gene has been reported with comparatively better results toward GB accumulation, overall protection in vegetative and reproductive parts and tolerance to multiple abiotic stresses (Sulpice et al., 2003; Park et al., 2004). Several transgenic plants with the *codA* expression exhibited multiple abiotic stress tolerance (**Table 1**). The usefulness of *codA* gene is evident from the fact that its expression under constitutive promoters exerted no penalties in terms of growth retardation (Park et al., 2007b). Rather, increased GB accumulation as a result of constitutive expression of *codA* in transgenic plants improved reproductive organs. Transgenic tomato plants with the *codA* gene showed chilling stress tolerance and increased fruit set by 10–30% (Park et al., 2007b). Further, the protective effects of GB were investigated on reproductive organs such as flowers and fruits (Park et al., 2007a). Transgenic tomato plants with constitutive expression of the *codA* gene exhibited large flowers and 54% heavier fruits compared to the non-transgenic control plants.

**Table 1:** Transgenic plants engineered with genes conferring abiotic stress tolerance.

Transgene	Source	Target plant	Tolerance	Physiological effect	Reference
*DREB1A*	*Arabidopsis thaliana*	*Arachis hypogaea*	Drought	Yield improvement of up to 24% in drought trials	Bhatnagar-Mathur et al., 2014
*codA*	*Arthrobacter globiformis*	*E. globulus*	Salt	–	Yu et al., 2009
*DREB1A*	*A. thaliana*	*G. max*	Drought	Improvement in number of seeds, number of pods	de Paiva Rolla et al., 2014
*AVP1*	*A. thaliana*	*G. hersutum*	Salt, drought	20% higher fibre yield in transgenic lines than that of wild-type under filed condition	Pasapula et al., 2011
*TaNHX2*	*T. aestivum*	*M. sativa*	Salt	High antiporters activity under 200 mM NaCl	Zhang et al., 2012
*AlNHX1*	*Aeluropus littoralis*	*N. tabaccum*	Salt	More Na^+^ accumulation in roots. High K^+^/Na^+^ ratio in shoots. About 150% increase in dry weight/plant	Zhang et al., 2008
*codA*	*A. globiformis*	*N. tabaccum*	Salt	–	Jingjia et al., 2013
*PgDREB2A*	*P. glaucum*	*N. tabacum*	Salt, osmotic	Fourfold higher germination at 200 mM NaCl. 50% higher seed germination under 400 mM mannitol	Agarwal et al., 2010
COX	*Arthrobacter pascens*	*O. sativa*	Salt	–	Su et al., 2006
*AtDREB1A*	*A. thaliana*	*O. sativa*	Drought	Improved rice spikelet (42% higher), grain yield (11% higher)	Xiao et al., 2009
*SbDREB2*	*Sorghum bicolor*	*O. sativa*	Drought	Significantly higher number of panicles	Bihani et al., 2011
*codA*	*A. globiformis*	*Solanum lycopersicum*	Chilling	–	Park et al., 2004
*codA*	*A. globiformis*	*S. lycopersicum*	Salt, drought	–	Goel et al., 2011
*codA*	*A. globiformis*	*Solanum tuberosum*	Salt, drought	–	Ahmad et al., 2008
*codA*	*A. globiformis*	*S. tuberosum*	Drought	–	Cheng et al., 2013
*TaDREB2/* *TaDREB3*	*T. aestivum*	*T. aestivum*	Drought	–	Office of the Gene Technology Regulator [OGTR], 2010
*AtDREB1A*	*A. thaliana*	*T. aestivum*	Drought	Improved WUE and acceptable yield under field conditions	Saint Pierre et al., 2012
*OsNHX1*	*O. sativa*	*Z. mays*	Salt	Increased biomass production	Chen et al., 2007

*(–) information are not known; WUE, water use-efficiency.*

### DREB-Transgenic Plants

*DREB* genes encode transcription factors which act as master switches to regulate the expression of many down-stream abiotic stress tolerance-responsive genes (Agarwal et al., 2006). An important observation of the *DREB*-transgenic plants was the associated stunted phenotype when expressed under constitutive promoters (Liu et al., 1998; Kasuga et al., 2004). However, the use of stress-inducible promoters to express *DREB* genes recovered the normal phenotype.

A limited number of transgeic plants engineered with DREB transcription factors have been evaluated and tested under realistic field conditions for their agronomic and yield performance under stress conditions. Some notable studies in several crops such as wheat, rice, peanut and soybean have revealed the effects of *DREBs* on several aspects of plant growth, stress tolerance and yield components (Xiao et al., 2009; Bihani et al., 2011; Saint Pierre et al., 2012; Bhatnagar-Mathur et al., 2014; de Paiva Rolla et al., 2014) (**Table 1**). Saint Pierre et al. (2012) conducted experiments on transgenic wheat lines with *AtDREB1A* gene to evaluate survival, recovery from stress as well as water use-efficiency under green-house conditions. Under these conditions, the transgenic lines performed well in terms of recovery from stress compared to control plants. Under field conditions, the transgenic lines did not outperform the control lines in terms of grain yield under drought stress. However, some transgenic lines which were selected for improved water use-efficiency had an acceptable yield even higher under well irrigated conditions. The authors concluded that although the transgenic lines did not show improved yield than control under stress condition, high yielding transgenic lines would be possible provided adequate transformation and screening protocols. Transgenic rice lines with sorghum *DREB2* gene were evaluated under water stress condition and showed a significantly higher number of panicles as compared to wild type plants under water stress condition (Bihani et al., 2011). Although the mean grain weight in both transgenic and wild type plants was the same under stress condition, the transgenic lines appeared to show improved yield due to an increase in the number of panicles rather than improved grain yield traits, the authors concluded. Bhatnagar-Mathur et al. (2014) reported significantly higher yield increase in transgenic *DREB1A* peanut lines under water stress condition in field experiments. This is the first report where the *DREB* transgenic lines showed a significant yield increase than wild type under stress conditions. Transgenic lines showed higher pod and seed yield than wild type under drought stress across all field trials. In another study, de Paiva Rolla et al. (2014) evaluated the agronomic performance of transgenic *DREB*-soybean lines under both greenhouse and field conditions. In these experiments, the transgenic lines did not outperform the wild type, but under drought stress, the transgenic lines showed improvement in some growth components such as number of seeds, number of pods with seeds and the total number of pods. The authors concluded that further studies to target full characterization of the soil and atmospheric conditions and interactions could result in transgenic plants that outperform the non-transgenic plants under stress conditions.

### Na^+^/H^+^ Antiporter Genes in GM Plants

In the wake of increasing soil and water salinization, the role of Na^+^/H^+^ antiporters is of tremendous importance. Over the last several years, transgenic plants with expression of antiporter genes demonstrated improved salt tolerance with or without the anticipated effects on plant growth and productivity (Khan, 2011b; Khan et al., 2015a). Some of the prominent results with antiporter genes are shown (**Table 1**). Transgenic plants expressing antiporter genes have shown promising results in cotton (Pasapula et al., 2011), maize (Chen et al., 2007), and tobacco (Zhang et al., 2008). In addition to the initial stress tolerance at the early plant growth stages with antiporter genes, durable stress tolerance may be achieved with positive effects on the overall plant growth and productivity under realistic field conditions. Strategies may involve regulation of antiporters specialized mechanisms in halophytes, use of superior alleles, and gene-stacking may be used (Khan et al., 2015a).

## Potential for Harm Associated with Candidate Abiotic Stress Tolerance Genes and Their Assessment

The regulatory decision making on the uses and deliberate environmental release of abiotic stress tolerance genes such as *codA*, *DREBs* and Na^+^/H^+^ antiporters depends on their safety to the environment and biodiversity. The environmental risk assessment of transgenic plants requires a case-by-case evaluation based on the information of the transgene, host plant and cultivation environment (Watanabe et al., 2005). The environmental risk assessment of these genes will be based on information on (1) specific nature of the gene, (2) type of stress tolerance, it confers, (3) specific plant phenotype, conferred by the gene, (4) underlying mechanisms controlled by these genes, (5) the nature of the host plant, and (6) characteristics of the potential receiving environment where the transgenic plants will be grown. Abiotic stress tolerance involves molecular, physiological and metabolic changes at the whole plant level. Therefore, the risk assessment process would focus on the whole plant and the potential receiving environment (Chaves et al., 2003).

In comparison to the insect resistance and herbicide tolerance genes, expression of abiotic stress tolerance genes could affect a wide range of plant growth and developmental functions (Chua and Tingey, 2006). As a result, the transgenic plants may show enhanced fitness advantage under selective pressure in agricultural and natural environment. This selective advantage may increase persistence and weediness potential in agricultural environment and may pose a challenge to the prevalent tillage and weed control practices (Warwick et al., 2009; Rudelsheim and Smets, 2010; BCH, 2011). The increased fitness advantage may have ecological impact by extending the spread of transgenic plants beyond their cultivation areas to natural environment (Chan et al., 2012). However, due to the limited available data on the ability of abiotic stress tolerance genes to confer enhanced fitness advantage, ecological impact assessment is difficult to predict (Chan et al., 2012). In addition, the risk assessment of abiotic stress tolerant transgenic plants may also encounter challenges such as choice of the comparator, behavior of the potential receiving environment and combination of selection pressures (Liang et al., 2014). Abiotic stress tolerance genes would require careful assessment as to what extent they confer fitness advantage and secondary effects and how it could be associated with weediness and invasiveness tendencies. With increasing number of transgenic plants entering field trials and environmental risk assessment studies, issues related to weediness and invasiveness would be easier to address.

### The Type of Transgene, Effects on Fitness, Weediness and Allelopathic Potential of Transgenic Plants

#### The *codA* Gene

The *codA* gene is isolated from the soil bacterium, *Arthrobacter globiformis*, and is salt tolerance-inducing in transgenic plants. The underlying mechanism of *codA* is production of GB that has a diverse role in plant tolerance and cellular protection from the damaging effects of salt stress (Khan et al., 2009). In some plants such as wheat and *Arabidopsis*, the GB application resulted in expression of genes which influenced diverse stress adaptation mechanisms (Allard et al., 1998; Einset et al., 2007). In addition, Kathuria et al. (2009) reported up-regulation of several stress responsive genes in the *codA* transgenic rice. The enhanced tolerance due to *codA* in transgenic plants might be attributed to the effects of GB and partly to other stress-related mechanisms. On the whole, the *codA* gene confers a selective advantage and improves plant phenotype under salt stress. The selective advantage and increased fitness in transgenic plants may confer enhanced volunteer and persistence potential in agricultural environment and invasiveness in natural environments (Lu, 2008). However, the *codA*-conferred stress tolerance and fitness advantage under stress condition may reduce yield losses of transgenic plant as compared to that of conventional plant. The selective advantage is limited under salt stress that may not change the persistence or volunteer potential of crop plants and their wild relatives. Nevertheless, the well-recognized principles for environmental risk assessment call for case specific consideration of potential for harm to the surrounding plant vegetation, rhizosphere microbial activities and ecological consequences if the transgene integrates in wild and weedy relatives of crop plants (**Table 2**).

**Table 2:** Environmental risks and likelihood of risks on the use of candidate abiotic stress tolerance genes in transgenic plants.

Transgene/protein	Potential hazard/risk	Likelihood of risks
*codA* (encoded protein/Glycine betaine)	Confers selective advantage, fitness, better plant growth may increase competitive potential of crop plants Allelopathic effect on surrounding plant vegetation and soil microbe diversity and functions Changes in salt tolerance may affect structure and functions of soil microbes	Selective advantage is limited, only under stress condition No competitiveness, weediness in crop plants No known adverse/allelopathic effects Metabolic changes, allelochemicals may have effects Unknown effects on rhizosphere microbes through changed salt tolerance, water and nutrients
*DREBs*/transcription factor proteins	Confers selective advantage under stress condition May have cross tolerance May have unintended effects These all factors may increase plant fitness	DREBs have no direct effects on plant diversity Selective advantage is limited and only under stress condition Cross tolerance may involve physiological, metabolic burdens, reduced fitness Increased fitness or differences in fitness and weediness traits, may not affect biodiversity May affect microbe diversity through changed soil abiotic condition
Na^+^/H^+^ antiporters	May confer selective advantage, improved phenotype Increased fitness may increase persistence and competitive ability of crop plants Selective advantage and improved phenotype may affect rhizosphere microbes and their functions	These genes and the encoded proteins are native to plants Selective advantage is limited that may not change persistence and volunteer potential Changed salt tolerance may have effects on soil microbes through changed water and nutrients

Allelopathic effect of the *codA-*encoded protein on the surrounding plant vegetation and soil microbes is an important element of the risk assessment process, practiced in some regulatory regimes in the world. The *codA-*encoded protein and the GB have no known direct effects on the surrounding plant vegetation, diversity of soil microbes and their enzymatic activities. Crop plants may not affect or compete with surrounding plant vegetation through allelopathic activity as these plants, mainly lack allelochemicals due to the process of domestication and selection (Leather, 1983). The potential impact of the *codA* gene on soil microbial communities and their enzymatic activities could be viewed in relation to the improved stress tolerance that may alter the transgenic plant capability to uptake water and nutrients from the soil. A number of factors have been reported in the literature which may influence the soil microbial diversity and their functions. These include factors such as changes in plant root exudates, type of plant, soil condition and plant physiological state (Bossio et al., 1998; Griffiths et al., 1999; Yang and Crowley, 2000; Butler et al., 2003; Green et al., 2007). While conducting environmental risk assessment of the *codA* gene, assessment of allelopathic effects on soil microbial communities and their activities would be element of the central focus. A more logical approach would be to first consider agronomic performance (improved phenotype) to determine if there is indeed a detectable change in water use and nutrient uptake (salt tolerance) by the *codA*-transgenic plant compared to that of the non-transgenic conventional counterpart.

#### The *DREB* Genes

The environmental concerns/risks associated with the use of DREB transcription factors are summarized (**Table 2**). DREB transcription factors are of plant origin and may not have direct adverse effects on the surrounding vegetation, rhizosphere microbes and non-target organisms. The DREBs trigger the expression of a large number of down-stream genes, working in different stress and developmental response mechanisms in plants (Agarwal et al., 2006, 2010). The resultant effects other than the intended stress tolerance may also include cross tolerance (Yoshioka and Shinozaki, 2009; Warwick et al., 2009) unintended effects both on plant metabolism and physiological profiles and on the overall phenotype of the plant (Ortiz et al., 2007; Chan et al., 2012). The intended stress tolerance, cross tolerance and associated unintended effects may increase fitness of the transgenic plants (Beckie et al., 2006; Beckie and Owen, 2007). The increased fitness may result into increased persistence, competitive ability and weediness potential under agriculture environment, and broader ecological impact, if the gene transfers to wild relatives (Bigelow, 2013). The underlying changes in plant metabolites and other unintended changes may also have the potential to affect soil microbes and their activities.

In comparison to other abiotic stress tolerance genes, *DREBs* may have broader impact due to regulation of the expression of a large number of stress-responsive genes that in turn may bring metabolic and physiological alterations. However, while using genes for stress tolerance in transgenic plants, the plant phenotype as the final product should be considered irrespective of the transgene type, the end products and the underlying physiological and metabolic changes. In addition, the increased fitness in transgenic crop plants is an intended trait and may not increase the competitive ability of crop plants as they lack such potential. In case of gene flow to wild relatives of crop plants, *DREBs* may have environmental and ecological risks (Lu, 2008). These risks may not arise due to two reasons. First, the cross tolerance due to *DREBs* may have metabolic and physiological burdens and the wild relatives may not show enhanced fitness advantage. Second, the other environmental factors may still regulate the survival and spread of these wild relatives. Moreover, fitness traits may not be confused with weediness traits, and the stress tolerance and fitness advantage due to *DREBs* may still fall within the natural range of varietal differences of crop plants for the trait. Many plants naturally contain *DREB* genes and adaptation to abiotic stresses may involve the same physiological and metabolic effects. Questions regarding the unintended effects in the form of transcriptomic or metabolomic changes have been raised but have never been reported based on current scientific knowledge and molecular tools (Ricroch et al., 2011; Simó et al., 2014). A few research studies have been conducted on the effects of selected transgenes (*ABF3*, *DREB1A* and mannose-6-phosphate reductase, *M6PR*) on transcritome profiles in drought and salt tolerant transgenic *Arabidopsis* (Abdeen et al., 2010; Chan et al., 2012). These studies concluded that transcriptome analysis reveals absence of unintended effects or it may be a poor predictor of secondary phenotypic or fitness effects in transgenic plants modified with salt and drought tolerance genes. Transcriptome profiling may be a helpful tool in the future to predict unintended effects, but may not be a substitute of the phenotypic comparison in the potential receiving environment.

Potential impact on rhizosphere microbial communities could be anticipated due to the multi-faceted role of *DREBs* on plant fitness. Alterations in plant phenotype and improved water use-efficiency due to *DREB* expression may potentially impact microbial community structure and their enzymatic activities. The rhizosphere microbial structural and functional diversity may be affected by a number of factors, including abiotic conditions (Bossio et al., 1998). This consideration could be of significance in the environmental risk assessment once it is established that the *DREB* expression in a particular plant increases water use-efficiency and nutrient uptake from the soil. After then, the meaningful differences may be used to draw hypothesis as to how the changed water use-efficiency could impact the microbial community structure and their activities.

#### Na^+^/H^+^ Antiporters

The antiporter genes and their encoded proteins are from the plant origin and have no known harmful effects. These proteins mediate transmembrane movement of Na^+^ and K^+^ ions; maintain cellular homeostasis and their overproduction may alter plant fitness under abiotic stress. The transgenic plants engineered with antiporters genes, so far, have not shown very high salt tolerance that may make them as strong competitors and may extend their range of cultivation to natural environment or making their wild relatives more invasive (**Table 2**). Conventional breeding approaches have utilized a natural variation at the intra-specific, inter-specific and inter-generic levels to develop salt tolerant varieties (Ashraf and Akram, 2009). Some of the developed varieties such as Alfalfa (Dobrenz, 1999) and bread wheat (Hollington, 2000) were tested under natural field conditions. It is obvious that these varieties might also have involved antiporter genes as major contributors to the conferred stress tolerance. Despite that, the risk assessment should deal these genes on a case-by-case basis, taking into account the biology of the parent plant and the potential receiving environment. The Na^+^/H^+^ antiporters-induced salt tolerance and improved phenotype may have effects on rhizosphere microbial diversity and functions through changed water and nutrient uptake. However, this consideration could only be taken in environmental risk assessment if transgenic plant outperforms the non-transgenic in terms of meaningful changes in agronomic characteristics, and improved water and nutrient uptake from the soil.

### The Type of Plant Engineered with Candidate Abiotic Stress Tolerance Genes

Based on the biological characteristics, plants have been distributed in three categories. These are domesticated crop plants, trees, and perennial grasses. Characteristics of these three groups have to be taken into consideration while assessing the environmental effects of the transgenes.

#### Domesticated Crop Plants

**FIGURE 1:**
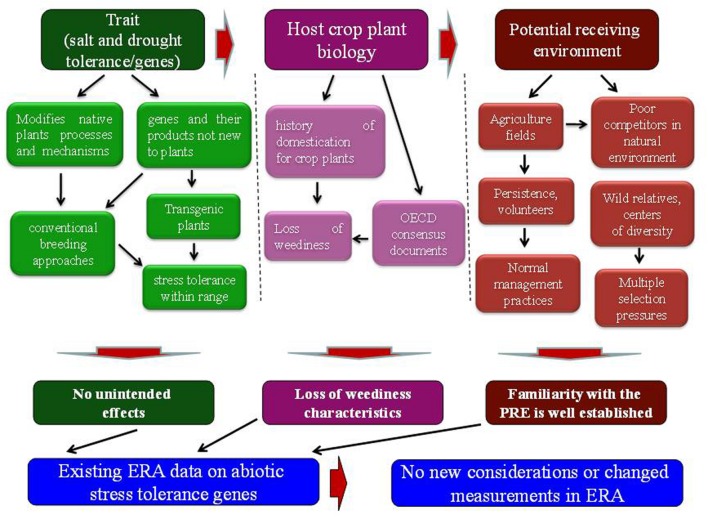
**Elements of ERA paradigm and needs for additional considerations.** The environmental risk assessment of transgenic plants requires information about the gene or trait, host plant biology and potential receiving environment.

Biology of the crop plant is well known. The crop plants have been passed through a long process of domestication, during which they have lost weediness characteristics (**Figure 1**). A number of OECD consensus documents are available on the biology of crop plants (Organization for Economic Cooperation and Development [OECD], 2012). According to these documents, crop plants have very low or negligible weedy characteristics and may not compete with grasses, trees and shrubs and also cannot establish as invasive in non-agricultural environments (Organization for Economic Cooperation and Development [OECD], 2006; Campbell and Snow, 2009). The improved abiotic stress tolerance and fitness advantage may not have an ecological impact because the improved plant growth and phenotype due to increased abiotic stress tolerance falls within the natural range of varietal differences for the stress tolerance trait. Conventional breeding approaches have long been used to exploit the varietal differences for genetic variation to improve abiotic stress tolerance in crop plants (Singh et al., 2014). Therefore, modification with fitness enhancing abiotic stress tolerance genes may not make them potential weeds or to cause them invasive in non-agricultural environments.

#### Trees

Tree plantation for both forest and non-forest purposes has been practiced for a long time. In the recent past, the tree plantation for forests has been increased by about 5 Mha annually during the period from 2005 to 2010 (FAO, 2010). This rapid increase in tree plantation was achieved under highly managed practices such as land preparation, fertilization, weed control and the use of improved genotypes (Evans and Turnball, 2004; Fox et al., 2007). In many respects, the silvicultural practices resemble those used for crop plants in agricultural environments.

Recently, some tree species have been engineered with genes conferring abiotic stress tolerance and these examples are reviewed (Gambino and Gribaudo, 2012; Osakabe et al., 2012). The important tree species with salt and drought tolerance traits are pines, poplars and eucalyptus (Kikuchi et al., 2006, 2009; Tang et al., 2007; Li et al., 2009; Yu et al., 2009, 2013a,b). Field trials and risk assessment studies have been conducted on some forest and non-forest trees in the USA, New Zealand, Australia and Japan and these information and data are available (APHIS, 2012; Japan Biosafety Clearing-House [J-BCH], 2012; NZEPA, 2012; Office of the Gene Technology Regulator [OGTR], 2012b).

Many of the trees for forest or non-forest plantation have been extensively studied with a lot of information generated on their biology (Organization for Economic Cooperation and Development [OECD], 2012). Consensus documents on the biology of tree species under the OECD include some important trees such as spruce, poplars, pines, white birch, douglas-fir, and fruit trees such as stone fruits, papaya and banana. In addition to the OECD documents, individual contributions from several countries are available for several tree species (Organization for Economic Cooperation and Development [OECD], 2006; Craig et al., 2008). These data and information are valuable sources and may also be used for the environmental risk assessment of transgenic trees with salt and drought tolerance genes. Moreover, much is known about the biology of the trees and the potential receiving environment where these are under cultivation (Haggman et al., 2013). Based on these facts, the overall information and data collected from field trials and ERA of stress tolerant transgenic crop plants are equally applicable to the ERA of transgenic trees modified with salt and drought tolerance genes.

#### Perennial Grasses

Perennial grasses have more weediness and invasiveness tendencies than annual food crops. Some of the important perennial grasses such as turfgrass, forages and biofuel grasses are the important contributors in agriculture development (Wang and Brummer, 2012). Some examples of biofuel grasses are switch grass, jatropha, joint reed and miscanthus. In the recent past, many of these perennial plants have been transformed with genes conferring drought and salt tolerance (Wang and Brummer, 2012). Some biofuel plant species are considered as weeds in some parts of the world (Low and Booth, 2007; Crosty, 2009), and these plants such as jatropha may have the potential to become invasive (Executive Secretary, 2007). Few members of these grasses such as miscanthus, switchgrass, Johnson grass and others are placed on the invasive and the noxious plant list in the United States (Low and Booth, 2007; Wang and Brummer, 2012). There have been concerns of increased weediness, invasiveness and gene flow to close wild relatives and broader ecological impact associated with genetic improvement of perennial grasses, particularly with fitness enhancing traits (Kausch et al., 2010). So far, there are no documents available from the OECD on the biology of these perennial grasses. Some species such as perennial rye-grass, bluejoint reed-grass and miscanthus have been documented by the OGTR and the USDA. As these plants are mostly outcrossing, gene flow and ecological impact as a result of fitness enhancing abiotic stress tolerance genes would be important in the environmental risk assessment.

### Potential Receiving Environment

The transgenic plants with these genes will be disseminated to agricultural fields as the potential receiving environment, affected by abiotic stress (**Figure 1**). Familiarity with the conditions of agricultural fields is well established. In case of any volunteers or persistence of transgenic plants, agricultural management practices would be used in the same way as those for conventional non-transgenic crop plants. Moreover, crop plants are poor competitors in natural environments and may not compete with wild plant populations (Sala, 2000). Wild relatives contain more genetic diversity than their domesticated crop plants (Jarvis et al., 2008). The adaptation process to environmental stresses seems to be much more complex in wild plant populations. In addition, multiple selection pressures exist in natural environment that regulate the spread of wild relatives. As a result of introgression, selective advantage to one or two stresses with a single gene may not confer fitness advantage to that level that could affect the spread and invasiveness of a wild relative (Kwit et al., 2011; Ellstrand et al., 2013). Unlike domesticated crop plants and trees, perennial grasses with fitness enhancing abiotic stress tolerance genes are more prone to pose weediness and invasiveness issues upon escape to the natural environment. Therefore, characteristics of the potential receiving environment need careful consideration in the risk assessment process.

## Examples of Field Trials and Risk Assessment Studies

A number of transgenic plants with genes conferring abiotic stress tolerance, particularly salt and drought are under field trials for environmental risk assessment studies (**Table 3**). Monsanto conducted risk assessment studies on transgenic maize with the *CspB* gene under drought stress (APHIS, 2011). In its first report regarding the evaluation data, the USDA concluded that the MON 87460 performs better than its conventional counterpart under limited water conditions. In addition, the transgenic maize is no different from the conventional maize in terms of weediness and invasiveness potential, cross-tolerance, other unintended/pleiotropic effects and overall ecological impact. Recently, Sammons et al. (2014) further characterized the MON 87460 and its conventional maize for agronomic and phenotypic data generation and subsequent use of the data for environmental risk assessment. The generated data were used to analyze the potential of transgenic maize for increased persistence, weediness, invasiveness, crossability, and other unintended effects that may collectively affect agricultural and natural environments. Based on the agronomic and phenotypic characterization, no significant and meaningful changes were found between the MON 87460 and its conventional counterpart that could affect persistence or volunteer potential. The transgenic maize behaved no different than its conventional maize except the intended trait of low yield loss under limited water conditions. They further concluded that the environmental risk assessment strategies which were used for insect resistance and herbicide tolerant plants are equally applicable to abiotic stress tolerant plants such as MON 87460.

**Table 3:** Examples of abiotic stress tolerant transgenic crop plants and trees under field trials for risk assessment studies.

Abiotic stress tolerance	Transgene	Host	Target crop plants/Trees	Implementing organization	Reference
Drought	*CspB*	*B. subtilis*	*Z. mays*	Monsanto	APHIS, 2011
Drought	*OsDREB1A*, *ZmDof1*	*O. sativa*, *Z. mays*	*S. officinarum*	BSES Limited, Aus	Office of the Gene Technology Regulator [OGTR], 2009
Drought	*TaDREB2/* *TaDREB* *AtAVP1*	*T. aestivum*, *A. thaliana*	*T. aestivum*, *H. vulgare*	University of Adelaide	Office of the Gene Technology Regulator [OGTR], 2010, 2012a
Drought	CCI	–	*T. aestivum*	VDPI	Office of the Gene Technology Regulator [OGTR], 2008a, 2013
Drought	*Asr1, PEPC*	*Z. mays*, *Sorghum*	*Z. mays*	Biogemma	SBC-Schenkelaars Biotechnology Consultancy (2007)
Salt	*Ornithine* *aminotransferase*	–	*T. aestivum*	Grain Biotech Aus	Office of the Gene Technology Regulator [OGTR], 2005
Salt	*codA*	*A. globiformis*	*E. camaldulensis*	Tsukuba University	Japan Biosafety Clearing-House [J-BCH], 2005; Kikuchi et al., 2009
Salt	*codA*	*A. globiformis*	*E. globulus*	Tsukuba University	Yu et al., 2013a,b
WUE/NUE	*AtMYB2*, *Zmdof1*	*A. thaliana*, *Z. mays*	*S. officinarum*	BSES Limited, Aus	Office of the Gene Technology Regulator [OGTR], 2007
WUE	CCI	–	*G. hirsutum*	Monsanto	Office of the Gene Technology Regulator [OGTR], 2008b
Water logging tolerance	*Adh*, *Pdc*	*G. hirsutum*, *A. thaliana*	*G. hirsutum*	CSIRO Aus	Office of the Gene Technology Regulator [OGTR], 2008c
Cold	*CBF2*	–	*Eucalyptus*	ArborGen	APHIS, 2012, 2009a,b

*ARC, Agriculture Research Council; BSES, Bureau of Sugar Experiment Stations; Aus, Australia; CCI, confidential commercial information; CSIRO, Commonwealth Scientific and Industrial Research Organization; WUE, water use-efficiency; NUE, nitrogen use-efficiency; VDPI, Victorian Department of Primary Industries; *Asr1*, abscisic acid stress ripening; PEPC, phosphoenolpyruvate carboxylase (Modified from Khan, 2011a).*

In Australia, risk assessment studies were conducted on several transgenic plants such as wheat, barley, sugarcane, maize, and cotton under abiotic stresses and the results were submitted to OGTR for further approvals. The OGTR risk assessment and risk management plan concluded that the transgenes in these plants confer selected advantage under stress condition. Both the transgenic and non-transgenic plants are equivalent under non-stress conditions. The selective advantage may not change other characteristics of the plant. However, any unintended pleiotrophic effects could be judged during the pre-trial stage or through further monitoring and containment measures. The RARMP concluded that for future large scale release, additional information regarding weediness characteristics such as tolerance to multiple abiotic stresses changed reproductive capacity and disease susceptibility will be required in the risk assessment.

One of the prominent examples is the risk assessment studies on transgenic eucalyptus tree with the *codA* gene for salt tolerance. Transgenic *Eucalyptus camaldulensis* and *Eucalyptus globules* showed salt tolerance under semi-confined conditions (Kikuchi et al., 2006; Yu et al., 2009). Several rounds of risk assessment studies have been completed on transgenic eucalyptus in the greenhouse and field levels. The effect of *codA* gene was determined on allelopathic potential, soil microbial activities, weediness and competitiveness potential and cross-ability. No significant effect was found between the transgenic and non-transgenic lines of *E. camaldulensis* for the tested parameters (Kikuchi et al., 2009). Similar results were found in *E. globules* when assessed for allelopathic potential and soil microbe investigations (Yu et al., 2008; Lelmen et al., 2009). In addition, a 4 years filed trial was carried out for transgenic *E. globules* with the *codA* gene and the non-transgenic plants to analyze the impact on biomass production, soil microbial communities and surrounding vegetation (Oguchi et al., 2014). No significant effect was found on the tested parameters between transgenic and non-transgenic lines. These results revealed that the salt tolerance conferred by the *codA* gene has no significant impact on environmental aspects under environmental risk assessment.

Transgenic eucalyptus hybrid clone (*Eucalyptus grandis* × *Eucalyptus urophylla*) engineered with the *CBF* transgene that confers cold/freeze tolerance is under vigorous field trials and risk assessment studies in the United States (Nehra and Pearson, 2011). The ArbrGen has submitted petitions for transgenic eucalyptus to the USDA/APHIS for interstate movement and field trials at various locations. Approvals have been granted for interstate movement and confined field trials. Based on the biological characteristics and the nature of the transgene and the conferred tolerance, the APHIS considered that it is unlikely that the introduced gene and the cold tolerance trait would make the eucalyptus tree as weedy or invasive (APHIS, 2009a,b, 2012). In addition, the APHIS concluded that the confined release would not affect biodiversity upon the transgenic eucalyptus trees reaching to maturity and flowering.

## Elements of ERA Paradigm and Need for Additional Considerations

Most of the abiotic stress tolerance genes including DREBs and Na^+^/H^+^ antiporters and the underlying stress tolerance mechanisms are not new to plants. Conventional breeding approaches which have been used to date have relied on the same abiotic stress tolerance genes and the underlying mechanisms. While considering the adverse ecological impacts, the nature of the novel phenotype is important irrespective of the method of modification either through conventional breeding or genetic engineering approaches (Wolt et al., 2015). In case of transgenic plants with abiotic stress tolerance, the magnitude of the conferred stress tolerance would be given the central focus as a potential hazard that may affect non-target organisms. The amount of stress tolerance could be checked by comparing the transgenic plant with a non-transgenic conventionally developed variety in the target environment. Therefore, environmental risk assessment should focus plant phenotype and the potential receiving environment rather than the nature of the introduced gene and the underlying mechanisms. Changes in agronomic performance and plant phenotype that could have an ecological impact needs careful consideration in the problem formulation step. The transgenic plants developed with abiotic stress tolerance genes so far have shown limited stress tolerance under greenhouse and field studies (**Tables 2** and **3**). In addition, the risk assessment studies conducted under the OGTR and Monsanto also reported limited stress tolerance in transgenic plants. The stress tolerance conferred these plants an overall growth and yield advantage that was prominent only under stress conditions. Therefore, the limited stress tolerance and fitness advantage may not have an ecological impact. Despite these few examples of risk assessment studies, the recently adopted trend of using gene stacking approach for more durable stress tolerance may result transgenic plants with fitness costs and benefits than their non-transgenic control plants (Londo et al., 2011). Uncertainties over the potential of increased stress tolerance and fitness advantage and the resulting ecological impact may be countered through continuous monitoring. Unlike food crops, the biofeedstock crops and the perennial grasses are expected to pose ecological concerns due to their comparatively more weediness tendencies and also an extension of their cultivation in marginal areas. While advancing environmental risk assessment for transgenic biofeedstock crops, Wolt (2009) mentioned that the ERA for these crops with abiotic stress tolerance genes should focus on the weediness and invasiveness related aspects. Here, the question arises whether enough information on weediness and invasiveness are available for biofeedstock perennial grasses, forages and biofuel plants? This question could be answered through revisiting the problem formulation step of risk assessment which has already been described for drought tolerant maize (Nickson, 2008). It could be further strengthened by putting increased information on the weediness and invasiveness potential of these plants and their phenotypic and agronomic characterization in the potential receiving environment. An appropriate comparative approach and sound analysis plan would be necessary to focus on key aspects related to persistence, weediness and invasiveness tendencies. Moreover, emphasis should be placed on identification of meaningful differences, prevalence of multiple abiotic stresses, choice of the comparator and response of the conventional plant to the target stress, optimal conditions and potential receiving environment.

## Conclusion

The environmental risk assessment process on candidate abiotic stress tolerance genes should be simple and straightforward and would take into account the long history of crop domestication, crop husbandry, agricultural management practices and natural variation of stress tolerance among crop varieties. Although a limited number of transgenic plants with abiotic stress tolerance genes have been evaluated for environmental risk assessment under field conditions, these studies have revealed no adverse effects of the transgenic plants to the environment and biodiversity. As more transgenic plants with abiotic stress tolerance genes enter field trials for agronomic performance and overall stress tolerance, more data will be generated that would help answer questions regarding uncertainties over the weediness and invasiveness issues. So far, in comparison to commercial traits such as insect resistance *BT* and *Ht* genes, the use of *codA*, *DREBs* and Na^+^/H^+^ antiporters do not need additional considerations or new and changed measurements in assessing the environmental effects of these genes. No specific assessment methodologies or techniques such as “omics” (transcriptome, proteome and metabolome analysis) are required to assess the increased fitness or related secondary effects. In regulatory decision-making on the deliberate environmental release of these genes, the final plant phenotype should be the prime target, not the transformation process and the diverse mechanisms that these genes may influence. Moreover, the use of these genes in transgenic plants and release into the environment should be considered on the risk-benefit-based analysis. In case of transgenic perennial grasses and biofeedstocks with more fitness enhancing abiotic stress tolerance genes, the environmental risk assessment would carefully consider the potential ecological impact.

## Author Contributions

MSK is the main author of this manuscript. He has contributed more than 80% to the technical and management of the manuscript. MAK checked the manuscript for technical as well as grammatical errors and helped to reshape the manuscript in final shape. DA helped in finding the relevant literature and manuscript preparation.

## Conflict of Interest Statement

The authors declare that the research was conducted in the absence of any commercial or financial relationships that could be construed as a potential conflict of interest.
